# Different Effects of Empagliflozin on Markers of Mineral-Bone Metabolism in Diabetic and Non-Diabetic Patients with Stage 3 Chronic Kidney Disease

**DOI:** 10.3390/medicina57121352

**Published:** 2021-12-11

**Authors:** Anna Masajtis-Zagajewska, Tomasz Hołub, Katarzyna Pęczek, Agnieszka Makówka, Michał Nowicki

**Affiliations:** Department of Nephrology, Hypertension and Kidney Transplantation, Medical University of Lodz, Central University Hospital, 92-213 Lodz, Poland; ania_pl@wp.pl (A.M.-Z.); tomasz.holub@gmail.com (T.H.); katarzyna.peczek90@gmail.com (K.P.); makag@wp.pl (A.M.)

**Keywords:** diabetic kidney disease, SGLT-2 inhibitors, calcium, phosphate, albuminuria, phosphatonins

## Abstract

*Background and objectives*: Treatment with sodium–glucose co-transporter 2 (SGLT2) inhibitors decrease tubular reabsorption of phosphate, which may explain the reduction of bone mineral density and an excess of bone fractures observed in some studies with this class of drugs. Since an increased risk of bone fractures may also be a result of diabetes itself, our study aimed to compare the effect of empagliflozin on the markers of mineral-bone metabolism between diabetic (DKD) and non-diabetic (ND-CKD) patients with stage 3 chronic kidney disease (CKD). *Materials and Methods*: Forty-two patients with stage 3 CKD and A2 albuminuria, including 18 with DKD and 24 ND-CKD, were investigated. All subjects received 10 mg empagliflozin for 7 days. Serum calcium, phosphate, parathormone (PTH), calcitriol, bone alkaline phosphatase (BAP), FGF-23 and urine calcium, phosphate, albumin and the renal tubular maximum reabsorption rate of phosphate to the glomerular filtration rate (TmP-GFR) were measured before and after empagliflozin administration. Differences in biomarkers response to empagliflozin between DKD and ND-CKD were the main measures of outcome. *Results*: There was a significant increase of PTH, FGF-23 and phosphate in DKD but not in ND-CKD whereas BAP and TmP/GFR did not change in either group. The reduction of albuminuria was only significant in ND-CKD. *Conclusions*: The effect of SGLT2 inhibitor on serum mineral and bone markers and on albuminuria in patients with CKD may be differently modified by the presence of diabetes mellitus.

## 1. Introduction

Disorders of bone and mineral metabolism (CKD-MBD) are one of the most prevalent and clinically relevant complications of chronic kidney disease (CKD) [1]. Impairment of phosphate excretion leads to phosphate retention, which is one of the earliest metabolic disorders in CKD. Phosphate retention triggers a secretion of phosphatonins, mainly fibroblast growth factor 23 (FGF-23) from osteocytes and parathyroid hormone (PTH), from the parathyroid glands [1].

Both high serum phosphate levels, high FGF-23 and deficiency of active vitamin D (calcitriol) are well-known non-classical risk factors for cardiovascular disease in both CKD patients and those without renal impairment [2,3,4,5,6].

Sodium–glucose cotransporters (SGLT) are membrane proteins involved in the transport of glucose, amino acids, vitamins, ions and osmolytes through the proximal tubule brush border and the intestinal epithelium [7]. The SGLT type 2 cotransporter is expressed in the kidney, most abundantly in the apical membrane of the proximal renal tubules, where it controls the reabsorption of glucose and sodium from the glomerular filtrate [8,9,10,11]. The kidneys are the main site of action of SGLT2 inhibitors, which selectively inhibit the sodium–glucose cotransporter. In addition to affecting glucose and sodium homeostasis, SGLT2 inhibitors induce several indirect effects on calcium and phosphate metabolism. The inhibition of proximal tubule reabsorption of Na^+^ and glucose increases Na^+^ availability in the regions expressing sodium-dependent phosphate cotransporters NaPi-IIa, NaPi-IIc and PiT-2 [12] resulting in increased phosphate reabsorption, which in turn leads to a reduction of urinary phosphate excretion [13,14,15].

It has been hypothesized that SGLT2 inhibitors, by increasing the serum concentration of phosphate, indirectly stimulate PTH production [16] and may also increase FGF-23 expression [17]. However, the mechanism by which phosphate regulate FGF-23 remains unclear [18]. In vitro studies have shown that phosphate has a direct effect on the expression of FGF-23 mRNA in primary human fetal bone cells [19]. Although, another study suggested that phosphate may decrease FGF-23 expression only through an indirect mechanism-possibly mediated by PTH [18].

Bone-specific alkaline phosphatase (BAP) is synthesized by the osteoblasts and is involved in the calcification of bone matrix. Its precise role in the bone formation process is unknown. Some studies have found an advantage of BAP over PTH in assessing bone turnover [20,21]. Bone-specific ALP also has a much longer half-life (1–2 days) compared to PTH (4 min) and shows less day-to day fluctuations [20,21].

Therefore, in contrast to PTH, which is not a direct marker of bone turnover, BAP as a product of osteoblasts is considered to be a highly specific marker of the bone-forming activity. In patients with advanced CKD, BAP has lower biological variability, making it a better alternative to serum PTH when monitoring mineral-bone disorder [22]. It has been postulated that the treatment with an SGLT2 inhibitor may lead to increased bone resorption and may increase the risk of bone fractures [16,18]. Increased levels of FGF-23 may contribute to this effect [18].

Most seminal studies evaluating the effect of SGLT2 inhibitors on calcium–phosphate metabolism in patients with chronic kidney disease were conducted only in patients with type 2 diabetes [23,24,25,26]. It is not clear whether the potential effect SGLT2 inhibitors would be limited to diabetic patients with CKD who are known to have an earlier onset and greater severity of CKD-MBD compared to non-diabetic CKD patients [27]. Our study was designed to directly compare the effect of an SGLT2 inhibitor empagliflozin on the changes of CKD-MBD biomarkers between diabetic and non-diabetic patients with CKD.

## 2. Materials and Methods

### 2.1. Patients

In this single-center, single-arm, prospective study 42 clinically stable patients (17 females, 25 males) with estimated glomerular filtration rate (eGFR) between 30–60 mL/min per 1.73 m^2^ and increased albuminuria were enrolled.

The participants were divided into 2 subgroups, i.e., 24 subjects with non-diabetic chronic kidney disease (ND-CKD) (11 females, 13 males, mean age 53.7 ± 9 years) and 18 patients with diagnosis of diabetic kidney disease (DKD) (6 females, 12 males, mean age 58.8 ± 8.4 years).

Study participants were recruited from among patients undergoing outpatient nephrological care who came for a routine visit. The main inclusion criteria were CKD stage 3 (2002 KDIGO Guideline) with a stable kidney function, i.e., eGFR ± 5% for at least 6 months prior to the study and were not expected to start the renal replacement therapy within the next 6 months, and category A2 albuminuria (30–300 mg/g creatinine). Patients with diabetic kidney disease must have been previously diagnosed with diabetic retinopathy and stable blood glucose control, i.e., glycated hemoglobin HbA1c < 8.0% in the last 3 measurements [28]. The patients with a history of kidney transplantation or receiving the medications that may affect calcium–phosphate metabolism (e.g., vitamin D, phosphate binders and anti-osteoporotic drugs) or an SGLT2 inhibitor anytime in the last 6 months were excluded. Exclusion criteria also included uncontrolled or treatment-resistant hypertension, chronic steroid therapy, chronic heart failure NYHA stage 3 or higher cardiovascular events in the last 6 months malignancy. The doses of all other medications were unchanged throughout the study.

The causes of chronic kidney disease in the non-diabetic patients included chronic glomerular disease in 16 subjects, autosomal dominant polycystic kidney disease in 3, hypertensive kidney disease in 3, and 2 patients had unknown causes.

The study protocol was approved by the Local Ethics Committee, and the study followed the principles of the Declaration of Helsinki. All participants were informed about the aim and design of the study and provided a written informed consent prior to the screening visit.

The study was supported by the Medical University of Lodz research Grant No. 503/1-151-02/503-01 and the study protocol has been registered in clinicaltrials.gov database (NCT04961931).

### 2.2. Methods

All subjects received detailed dietary recommendations at the time of enrollment and were advised to keep a diary of their meals. They were asked to have three meals a day during the study containing 0.6 to 0.8 g protein/kg/day and less than 700 mg phosphate/day based on recommendations of the Food and Nutrition Committee of the Institute of Medicine [29].

During 7 days of the study all subjects received oral empagliflozin 10 mg once daily in the morning.

Serum calcium, phosphate, parathormone, bone-specific alkaline phosphatase, fibroblast growth factor 23, plasma calcitriol, and urine calcium, phosphate and albumin were measured at baseline and after 7 days of empagliflozin treatment. Blood samples were obtained after an overnight fasting and collected in EDTA and serum tubes. The samples were immediately centrifuged at 3000 mm in 4 °C for 10 min and supernatant frozen at −80 °C until batch analysis. Plasma FGF-23 was measured with ELISA (Merck Millipore, St. Louis, MO, USA). Plasma 1.25(OH)_2_D was measured using a direct ELISA kit (ImmunDiagnostic, Bensheim, Germany). Urine samples were taken from the first morning portion of urine for the determination of albumin, calcium, phosphate and creatinine.

Serum iPTH was determined with a chemiluminescence assay using intact PTH reagent kit (ARCHITECT i2000SR; Abbott Diagnostics, Abbott Park, IL, USA)). Serum calcium, phosphate, creatinine and HbA1c as well as urine calcium, phosphate, and creatinine were measured with standard automated methods in our local laboratory. Estimated GFR was calculated using the creatinine-based CKD-EPI equation according of 2012 KDIGO recommendation [30].

Fractional excretion of calcium (FEca) was calculated using the standard equation: FEca = ([urine calcium] × [serum creatinine])/([serum calcium] × [urine creatinine]). Fractional excretion of phosphate was calculated as: FEP = ([urine phosphate] × [serum creatinine])/([serum phosphate] × [urine creatinine]). Tubular fractional reabsorption of phosphate (TRP) was calculated as TRP = 1-([urine phosphate] × [serum creatinine])/([serum phosphate] × [urine creatinine]) and percent TRP was calculated by multiplying TRP by 100. The tubular maximum reabsorption of phosphate per glomerular filtration rate (TmP/GFR [mmol/L]) can be calculated by the following recommendation [31]: If TRP is ≤ 0.86 (86%), then TmP/GFR = TRP × (serum phosphate). If TRP is > 0.86 (86%), then TmP/GFR = (0.3 × TRP)/(1 − 0.8 × TRP) × (serum phosphate) [32].

The results are presented as an arithmetic mean ± SD or median and interquartile range (IQR) for normally and non-normally distributed variables, respectively. The normality of each variable distribution was checked by a Kolmogorov–Smirnov test. Categorical variables were analyzed with chi-square test. Depending on the continuous variable distribution, t-test or Mann–Whitney U-test was used to compare means between two independent subgroups. Paired t-test or Wilcoxon’s test was used for dependent variables. *p* value < 0.05 was taken as significant. Pearson’s or Spearman’s correlation coefficients were calculated to assess relations between two variables depending on the normality of data distribution. In a linear multiple regression analysis, the change of TRP during empagliflozin administration was a dependent variable and diabetic status was a grouping variable. The statistical analysis was performed, and graphs were plotted, using the Statistica software, version 13.0 PL.

## 3. Results

**Figure 1:**
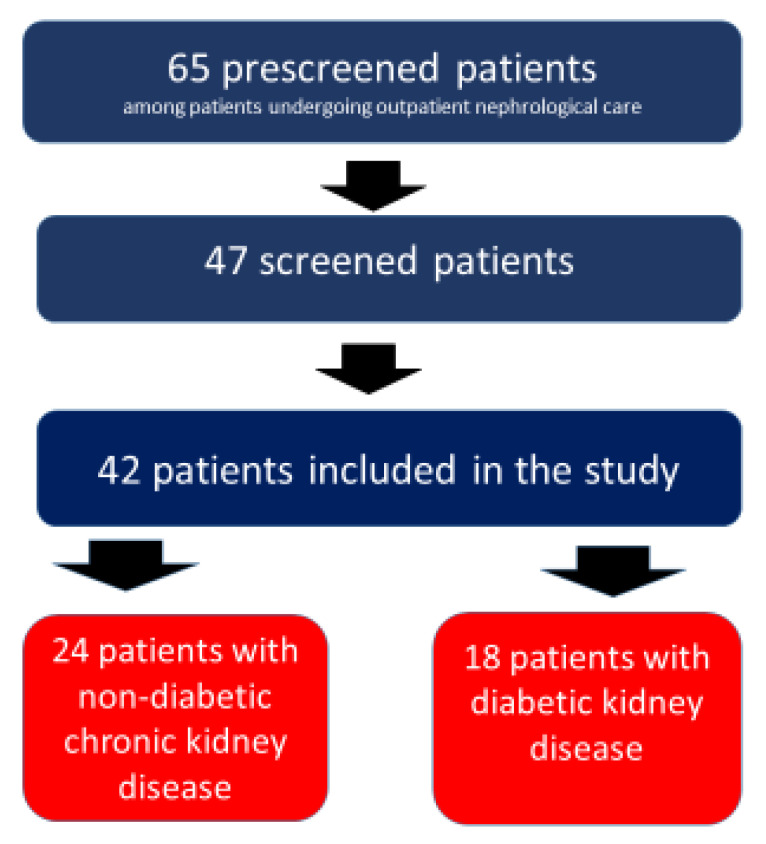
Flowchart of patients.

Forty seven patients were initially screened but five of them decided not to take part in the study after familiarizing themselves with the study protocol. As a result, 42 patients completed the study according to the protocol (Figure 1).

The baseline clinical and biochemical characteristics of the study participants are presented in Table 1. There were no significant differences in sex distribution, age and eGFR between DKD and ND-CKD subgroups. Baseline mean values of serum calcium, phosphate, 1.25(OH)_2_D, FGF-23, BAP, TRP, TmP/GFR, fractional excretion of phosphate and fractional excretion of calcium also did not differ significantly between the subgroups.

Serum PTH was significantly higher in diabetic patients (*p* = 0.02). Mean HbA1c value at screening in patients with DKD was 6.6 ± 0.2% (range 5.9–7.9%). Serum levels of calcium, phosphate, PTH, FGF-23, BAP, plasma 1.25(OH)_2_D and urine calcium, phosphate, TRP, Tmp/GFR, fractional excretion of phosphate and calcium before and after 7-day administration of empagliflozin are presented in Table 2.

Serum phosphate concentration in the ND-CKD group during empagliflozin administration did not change. However, in DKD patients, serum phosphate concentration significantly increased after the treatment (*p* = 0.02).

In DKD subgroup but not in ND-CKD serum PTH concentration was significantly higher after the treatment compared to baseline (*p* = 0.0016).

PTH levels did not differ between DKD and ND-CKD subjects neither before nor after the administration of empagliflozin. The serum FGF-23 concentration was higher only in the DKD group after the treatment with empagliflozin than at baseline (*p* = 0.03).

Plasma 1.25(OH)_2_D_3_ and serum BAP did not change after the treatment in the whole study group and in the subgroups. Urine phosphate and calcium did not significantly change during the study.

Renal tubular reabsorption of phosphate as well as their fractional excretion did not change after empagliflozin therapy in any group.

**Figure 2:**
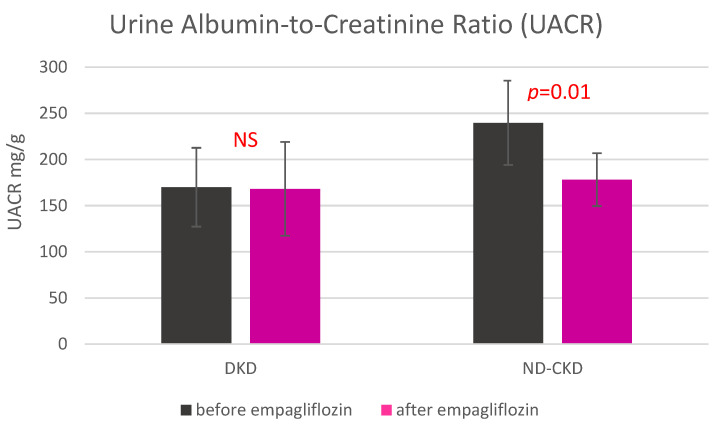
Urine albumin-to-creatinine ratio before and after the 7-day treatment with empagliflozin in diabetic and non-diabetic patients with chronic kidney disease. Abbreviations: UACR—urine albumin-to-creatinine ratio, DKD—diabetes kidney disease, non-DKD—non diabetes kidney disease.

Urine albumin-to-creatinine ratios (UACR) in ND-CKD and DKD subgroups are shown in Figure 2. The UACR significantly decreased in non-DKD but not in DKD subgroup.

During empagliflozin treatment, there were significant positive linear correlations between the changes of serum FGF-23 and TmP/GFR (r = 5174, *p* < 0.001), BAP and TmP/GFR (r = 9587, *p* < 0.001) and PTH and TmP/GFR (r = 6876, *p* < 0.001) in the whole study group positive linear correlations were also observed between BAP and TmP/GFR both before and after empagliflozin therapy in both subgroups, i.e., in ND-CKD: (r = 8681, *p* < 0.001 and r = 8596, *p* < 0.001, respectively), and in DKD (r = 9532, *p* < 0.001 and r = 9480, *p* < 0.001, respectively).

In a multiple regression analysis in which the change of TRP during empagliflozin treatment was a dependent variable, three variables: serum PTH (beta = −0.65), BAP (beta = 0.76) and 1.25OH_2_D (beta = −0.47) at baseline explained significant parts of variation of the dependent variable only in diabetic patients (multiple R = 0.73 (*p* < 0.001), F = 3.10, R^2^ = 0.54).

## 4. Discussion

The main finding of our study was that the effect of an SGLT2 inhibitor on markers of bone mineral metabolism and glomerular damage in patients with CKD is differently modulated by diabetes status.

Since bone mineral parameters were rarely studied in patients receiving SGLT2 inhibitors, we were only able to compare our results with the results of a few other studies in which different SGLT2 inhibitors were administered, and the subjects differed in renal function and diabetes incidence. Blau et al. [33] conducted a randomized, controlled cross-over study in which 25 healthy volunteers received either canagliflozin 300 mg/d or a placebo for 5 days to compare their effects on FGF-23-1.25-dihydroxyvitamin D-PTH axis. This study demonstrated an increase of renal reabsorption of phosphate as early as after 2–4 h post oral dose of canagliflozin. SGLT2 inhibitor administration caused a reduction of urinary phosphate excretion and thus to an increase of serum phosphate. Additionally, calcium excretion increased slightly but without causing any changes of serum calcium. The authors hypothesized that an increased calcium excretion could be associated with the high tubular flow as a result of osmotic diuresis, thereby reducing paracellular calcium reabsorption in the proximal tubule and reducing the calcium gradient between the tubule and the medullar interstitium [34].

Contrary to Blau et al. [33], we did not observe a significant change of fractional tubular reabsorption of phosphate or changes of urinary calcium excretion after the administration of an SGLT2 inhibitor.

Some studies showed that FGF-23 was positively associated with TmP/GFR [35]. Because kidney disease leads to a loss of nephrons and a reduction in GFR, TmP/GFR is a better measure than plasma phosphate concentration alone for the quantitative monitoring of the recovery of tubular function [36].

We revealed positive correlations between the changes of serum FGF-23 concentrations and TmP/GFR. On the other hand, Komo et al. reported that serum FGF23 was not correlated with phosphate, corrected calcium, or TmP/GFR in patients with normal renal function [37]. The same was seen in our study, although our patients had an impairment of renal function. The study of Ozeki et al. [38], which included the patients without reduced kidney function and proteinuria, showed that association between FGF-23 and TmP/GFR may be weaker in the absence of renal dysfunction [38]. In our study after 7 days empagliflozin therapy, no significant changes of TmP-GFR were seen.

All our patients had moderate (stage 3) chronic kidney disease and A2 albuminuria which is typically associated with increased levels of phosphaturic hormone at baseline and in particular, FGF-23, for which rise is usually seen ahead of the increase of serum PTH and phosphate, and the decrease of 1.25 (OH)_2_D [1].

It is notable that until recently almost all clinical studies with SGLT2 inhibitors were limited to the patients with diabetes or diabetic kidney disease. In our study in which diabetic and non-diabetic patients with CKD were directly compared with the changes of serum phosphate, FGF-23 and PTH after the administration of empagliflozin were seen only in the former. Serum calcium and plasma 1.25 (OH)_2_D remained, however, unchanged. Interestingly, in our study the variability of TRP change after an SGLT2 inhibitor was explained in a linear regression model not only by PTH and calcitriol but also by BAP; this was only found in diabetic patients. It may suggest that empagliflozin may regulate the mineral metabolism in a more complex way in diabetic than in non-diabetic kidney disease.

In healthy volunteers, canagliflozin induced a prompt increase of serum phosphate 36 h after the drug administration, which triggered an increase of plasma FGF-23 and PTH with a simultaneous decrease of 1.25-dihydroxyvitamin D level within 24 h after initiation of canagliflozin [33]. An increase of serum phosphate and FGF-23 and a decrease of 1.25(OH)_2_D were only transient, and these parameters returned to baseline values 120 h after the dose of the SGLT2 inhibitor [33]. It seems that the increases of PTH and FGF-23 seen in our study were most likely a compensatory response to disturbed phosphate homeostasis [39,40]. Higher FGF-23 levels, in turn, may lead to the suppression 1.25(OH)_2_D through an inhibition of CYP27B1 and stimulation of CYP24A1 [41,42].

Weir et al. [15] conducted a study in diabetic patients with reduced eGFR and showed that 26-week canagliflozin administration was associated with only a small change of serum calcium and phosphate. Similar small increases of serum phosphate were reported in a study with dapagliflozin in diabetic patients [43,44]. The association of SGLT2 inhibitor treatment with an increase of serum phosphate was also confirmed in several large studies in diabetic patients, although its mechanism and clinical relevance remains unclear [15,45,46,47,48]. Our study was of short duration and the increase of serum phosphate remained at the end of the 7-day treatment period.

These findings may be relevant to the patients with CKD. In a post-hoc analysis of the IMPROVE trial in CKD patients with diabetes and albuminuria, the effect of 6-week dapagliflozin therapy was compared to a placebo [49]. The study showed significant increases of serum phosphate, FGF-23 and PTH but, interestingly, failed to show any effect on serum calcium concentration. However, there was a significant simultaneous decrease of plasma 1.25(OH)_2_D that may explain the lack of the effect of serum calcium. In our study, we did not see any changes of plasma 1.25(OH)_2_D but the treatment period of 7 days may have been too short to observe any changes in the plasma level of steroid hormones.

In our study, serum BAP concentration, which is a specific marker of osteoblast function, did not significantly change. It is corroborated by the finding of Watanabe et al. [50], but their study was conducted in a different population of patients i.e., with nonalcoholic fatty liver disease.

Serum calcium and plasma 1.25(OH)_2_D were not affected by empagliflozin in our study similar to several previous studies with SGLT2 inhibitors [43,51,52]. However, it should be noted that the effect of SGLT2 inhibitors, in particular on serum calcium, was not uniform as some previous studies showed a slight increase of serum calcium during the treatment [15,53].

Diabetes in itself is accompanied by the disturbances of the hormones that regulate bone metabolism. In addition, the activity of osteoblasts can be inhibited by hyperglycemia or hypoglycemic medications [54,55,56,57,58].

SGLT2 inhibitors have had an excellent safety record also in the mild-to-moderate CKD population [28]. The effect of these drugs on the increase of serum phosphate in CKD is important as higher serum phosphate concentrations were associated with higher mortality in CKD [59,60]. DKD patients are particularly susceptible to the toxicity of phosphate [59]. Increased serum FGF-23, which is the principal regulator of phosphate homeostasis in CKD, was also associated with worse kidney and cardiovascular outcomes [61].

We also made another interesting observation. The significant reduction of albuminuria was seen only in non-DKD patients and not in diabetic subjects. It may be due to a short duration of our study because the lowering effect of SGLT2 inhibitors on albuminuria has been well-confirmed in large trials in DKD patients [62,63,64,65,66,67,68].

The limitations of our exploratory study was that it was not randomized and blinded and was conducted in a small group of patients. Future studies should involve a reference group of healthy individuals or individuals with diabetes without renal impairment or microalbuminuria. An advantage of our study is that the diet was well-controlled and all subjects received detailed dietary recommendations at the time of enrollment.

In conclusion, our pilot study showed that short-term empagliflozin administration induces a significant effect on several specific markers of mineral metabolism, including serum phosphate, PTH and FGF-23, but this effect was seen only in the diabetic patients with CKD. In contrast, a significant effect of empagliflozin on a decrease of albuminuria was observed in CKD patients without diabetes. The longer persistence of these initial and preliminary effects and their potential clinically relevant effects in patients with more advanced CKD will require further research.

## Figures and Tables

**Table 1:** Baseline clinical and laboratory characteristics of all patients and the patients with diabetic and non-diabetic chronic kidney disease.

	All Patients (*n* = 42)	Non-Diabetic (*n* = 24)	Diabetic (*n* = 18)	*p* Value (Non-Diabetic vs. Diabetic)
Men/Women	25/17	13/11	12/6	NS
Age, years	55.9 ± 9	53.7 ± 9	58.8 ± 8.4	NS
eGFR _CKD-EPI,_ ml/min per 1.73 m^2^	38.6 ± 6.3	38.5 ± 6.3	38.8 ± 7.7	NS
HbA1c, %			6.6 ± 0.2	
Serum calcium (Ca), mmol/L	2.1 ± 0.02	2.1 ± 0.08	2.1 ± 0.30	NS
Serum phosphate (PO_4_), mmol/L	1.3 ± 0.08	1.3 ± 0.1	1.2 ± 0.20	NS
Serum PTH, pg/mL	43.4 ± 20.5	34.6 ± 2.6	54.8 ± 27.1	0.02
Plasma 1.25(OH)_2_D, pg/ml	27.7 ± 8.3	26.4 ± 11.7	29.4 ± 22.4	NS
Serum FGF-23, pg/mL	107.2 ± 34.9	106.2 ± 6.4	108.4 ± 87.1	NS
Serum BAP, µg/L	19.5 ± 3.3	17.3 ± 11.3	22.2 ± 1.7	NS
Serum albumin, g/L	33.6 ± 3.04	34.5 ± 6.6	32.3 ± 0.2	NS
Urine-Ca/creatinine, mmol/g	0.6 [0.2, 0.9]	0.6 [0.3, 0.9]	0.5 [0.3, 1.1]	NS
Urine-PO_4_/creatinine, mmol/g	15.7 ± 2.3	15 ± 14.7	16.6 ± 1.2	NS
TRP, %	67.6 ± 2.9	68.9 ±11.6	65.8 ± 17.3	NS
TmP/GFR, mmol/L	0.77 ± 0.14	0.80 ± 0.12	0.74 ± 0.06	NS
Fractional excretion of phosphate, %	32.4 ± 2.9	31.1 ± 11.6	34.1 ± 1.7	NS
Fractional excretion of calcium, %	0.5 [0.2, 1.0]	0.6 [0.2, 1.2]	0.4 [0.2, 0.8]	NS
Urine-albumin/creatinine, mg/g	209.0 ± 31.4	239.6 ± 45.6	169.9 ± 42.7	NS

Mean ± SD or Median [IQR]. Abbreviations: PTH-Parathormone, FGF-23—fibroblast growth factor 23, BAP—bone alkaline phosphatase, HbA1c—glycated hemoglobin, eGFR—estimated glomerular filtration rate, Ca—calcium, PO_4_—phosphate, TRP—Tubular fractional reabsorption of phosphate, TmP/GFR—tubular maximum reabsorption of phosphate per glomerular filtration rate.

**Table 2:** Effect of empagliflozin on serum and urine parameters of calcium–phosphate and bone metabolism in all subjects and in subgroups of diabetic and non-diabetic patients with chronic kidney disease.

Parameter	Subjects	Before Empagliflozin	After Empagliflozin	*p*-Value after vs. before Empagliflozin Administration
Serum total calcium (mmol/L)	All (*n* = 42)	2.1 [2.0, 2.3]	2.13 [2.1, 2.34]	NS
	ND-CKD (*n* = 24)	2.1 ± 0.08	2.2 ± 0.1	NS
	DKD (*n* = 18)	2.1 ± 0.3	2.1 [2.0, 2.2]	NS
Serum phosphate (mmol/L)	All (*n* = 42)	1.2 [1.1, 1.5]	1.2 [1.1, 1.34]	0.02
	ND-CKD (*n* = 24)	1.24 [1.1, 1.5]	1.25 [1.0, 1.39]	NS
	DKD (*n* = 18)	1.1 ± 0.1	1.2 ± 0.2	0.02
PTH (pg/mL)	All (*n* = 42)	34.7 [18.7, 57.8]	34.7 [18.7, 57.8]	NS
	ND-CKD (*n* = 24)	21.3 [14.5, 57.8] *	21.3 [14.55, 52.9]	NS
	DKD (*n* = 18)	54.8 ± 27.1	62.9 ± 43.2	0.0016
1.25(OH)_2_D (pg/mL)	All (*n* = 42)	27.2 [18.7, 36.7]	24.8 [20.1, 39.4]	NS
	ND-CKD (*n* = 24)	26.4 ± 11.7	26.8 ± 13.3	NS
	DKD (*n* = 18)	29.4 ± 22.4	13.8 ± 17.7	NS
FGF-23 (pg/mL)	All (*n* = 42)	98.4 [75.6, 130.3]	98 [75.6, 130.3]	NS
	ND-CKD (*n* = 24)	106.2 ± 6.4	98.7 [76.7, 128.7]	NS
	DKD (*n* = 18)	98.3 [76.4, 128.3]	98.3 [76.4, 128.3]	0.03
BAP (µg/L)	All (*n* = 4 2)	19.5 [12.9, 24.7]	20.3 [14.3, 25.8]	NS
	ND-CKD (*n* = 24)	13.6 [10.4, 23.8]	15.8 [11.8, 22.0]	NS
	DKD (*n* = 18)	22.2 ± 1.7	22.5 ± 1.4	NS
Urine-Ca/Cr, (mmol/g)	All (*n* = 42)	0.6 [0.2, 0.9]	0.42 [0.26, 0.93]	NS
	ND-CKD (*n* = 24)	0.6 [0.3, 0.9]	0.5 [0.33, 1.4]	NS
	DKD (*n* = 18)	0.5 [0.3, 1.1]	0.28 [0.22, 0.8]	NS
Urine-PO_4_/Cr, (mmol/g)	All (*n* = 42)	1.94 [1.5, 2.3]	1.8 [1.4, 2.2]	NS
	ND-CKD (*n* = 24)	1.9 ± 0.09	1.9 ± 1.3	NS
	DKD (*n* = 18)	2.0 ± 0.02	1.7 [1.5, 2.1]	NS
TRP, %	All (*n* = 42)	67.6 ± 2.9	64.5 ± 4.9	NS
	ND-CKD (*n* = 24)	68.9 ±11.6	66.5 ± 42.1	NS
	DKD (*n* = 18)	65.8 ± 17.3	61.8 ± 2.0	NS
TmP/GFR, mmol/L	All (*n* = 42)	0.75 [0.6, 0.9]	0.86 [0.68, 1.0]	NS
	ND-CKD (*n* = 24)	0.80 ± 0.12	0.92 [0.66, 1.0]	NS
	DKD (*n* = 18)	0.74 ± 0.06	0.83 ± 0.28	NS
Fractional excretion of phosphate, %	All (*n* = 42)	31.5 [21.2, 41.9]	29.2 [19.9, 42.8]	NS
	ND-CKD (*n* = 24)	31.1 ± 11.6	33.4 ± 42.2	NS
	DKD (*n* = 18)	34.1 ± 1.7	26.6 [20.4, 41.7]	NS
Fractional excretion of calcium, %	All (*n* = 42)	0.5 [0.2, 1.0]	0.4 [0.2, 1.0]	NS
	ND-CKD (*n* = 24)	0.6 [0.2, 1.2]	0.7 [0.2, 1.3]	NS
	DKD (*n* = 18)	0.4 [0.2, 0.8]	0.4 [0.2, 0.7]	NS

Mean ± SD or median [IQR]. Abbreviations: PTH—Parathormone, FGF-23—fibroblast growth factor 23, BAP—bone alkaline phosphatase, Ca – calcium, Cr—creatinine, PO_4_—phosphate, TRP—tubular fractional reabsorption of phosphate, TmP/GFR—tubular maximum reabsorption of phosphate per glomerular filtration rate. * *p* < 0.05 ND-CKD vs. DKD.

## Data Availability

The data presented in this study are available on request from the corresponding author. The data are not publicly available due to privacy.
